# Fine needle biopsy of malignant tumors of the liver: a retrospective study of 624 cases from a single institution experience

**DOI:** 10.1186/s13000-020-00965-5

**Published:** 2020-05-06

**Authors:** Lin Zhang, Zhenjian Cai, Joe Rodriguez, Songlin Zhang, Jaiyeola Thomas, Hui Zhu

**Affiliations:** Department of Pathology and Laboratory Medicine, University of Texas Health Science Center at Houston, 6431 Fannin St, MSB, 2.290, Houston, TX 77030 USA

**Keywords:** Liver metastases, Primary liver neoplasms, Fine-needle biopsy (FNB), Carcinoma of unknown primary (CUP), Hepatocellular carcinoma (HCC)

## Abstract

**Background:**

Liver is one of the most common organs involved by metastatic neoplasms. In addition, a number of primary tumors can arise in the liver. Fine needle biopsy (FNB) is the most commonly used method for diagnosis of liver masses. Not much literature is available during the past 10 years about FNB of liver tumors. All large studies were performed more than 15 years ago. With the introduction of new disease entities, new tumor classification systems, and new diagnostic methods, updated documentation of FNB of liver neoplasms is much needed.

**Methods:**

Liver FNB cases that were diagnosed as “Positive for Malignancy” between 2010 and 2018 were retrieved from the cytopathology database in our institution. Patient medical records, cytopathology and surgical pathology reports, and slides from selected cases were retrieved and reviewed.

**Results:**

Over 30 different types of malignant tumors were identified in 624 malignant FNB cases, with the most common tumors being metastatic colorectal and pancreatic adenocarcinomas. Rare tumors include EBV-positive leiomyosarcoma, mesothelioma, and paraganglioma, among others. A subset of patients presented with widespread metastases involving liver with no known history. Identifying the primary sites in those cases can be challenging. We also found that in our practice, a significant number of hepatocellular carcinoma were diagnosed by FNB in recent years.

**Conclusions:**

A tremendous variety of neoplasms can occur in liver. Accurate diagnosis is essential for proper patient management. Familiarization with morphological features and judicious usage of ancillary studies are essential for accurate diagnosis.

## Introduction

Fine needle biopsy (FNB) has long been established as an accurate and safe procedure for tissue diagnosis of liver masses. FNB of liver masses can be performed percutaneously or endoscopically under ultrasound or computed tomography (CT) guidance. The sensitivity and specificity of FNB for detection of liver malignancy are up to 94 and 100%, respectively [1–5]. False positives are rare, and false negative diagnoses are most often the result of sampling error. The main contraindications for liver FNB are uncorrectable bleeding diathesis, a lack of safe access route, and uncooperative patients. With modern-day techniques, complications are uncommon, including bleeding and very rare needle track seeding [6–10].

There is much debate during the past two decades regarding the use of FNB for the diagnosis of hepatocellular carcinoma (HCC). Advances in imaging techniques have made CT and MRI the standard diagnostic modalities for establishing an HCC diagnosis. Histopathologic confirmation is frequently not required prior to treatment [11–13]. In addition, the risk of needle track seeding is a rare but significant complication of FNB [9, 10]. However, radiological imaging does not always allow precise characterization of the tumor, especially for small HCCs (< 2 cm) [14]. Furthermore, the risk of needle track seeding can be reduced using a small gauge, non-cutting needle [8]. With the increasing use of personalized targeted molecular therapy where tumor tissue is required for molecular signature studies, FNB can provide valuable information for the management of patients with liver tumors, especially HCC [12, 13]. In recent years, we have seen a significant number of FNB for diagnosing HCC preoperatively.

Liver is one of the organs where metastases are more common than primary tumors [3]. Virtually any malignancy can metastasize to the liver. The most common presentation for metastatic tumors is the presence of multiple small liver nodules in patients with known history of malignancy. Solitary metastases occur only in 6% of all metastases to the liver. Large (more than 5 cm in size) solitary metastases are rarer, and can be difficult to distinguish from primary neoplasms clinically [15]. HCC is the most common primary liver malignancy and usually arises in a background of cirrhosis. Other primary malignant tumors include cholangiocarcinoma, and very rarely, primary angiosarcoma, lymphoma, and neuroendocrine tumor, among others [15].

A subset of patients present with high grade unclassifiable metastatic disease to the liver without a known primary (carcinoma of unknown primary/CUP). The primary site cannot be determined despite combined clinical, radiological and pathologic efforts. Recent advances in immunohistochemical techniques have identified lineage specific antibodies that allow accurate identification of the primary site in an increasing number of cases. However, in over 85% of patients with CUP, the primary site remains unknown despite an extensive workup and collaboration between clinical, radiology, and pathology teams [16, 17]. For unclassifiable high grade CUP, patients often present with widespread disease. The tumors lack morphologic differentiation and express limited markers for tumor classification. Patients with CUP have limited treatment options and very poor prognosis, with an average survival of several months. The prognosis for patients with unclassifiable high grade CUP is even worse.

Not much literature is available during the past 10 years about FNB of liver tumors. All large studies were performed more than 15 years ago [1, 4, 5, 7]. The landscape of pathology is constantly changing with the introduction of new disease entities, new diagnostic methods and techniques, and new tumor classification systems including soft tissue tumors, head and neck tumors and neuroendocrine neoplasms, etc. [18, 19] . An updated documentation of FNB of liver neoplasms is much needed. In this study, we retrospectively reviewed 624 malignant tumors diagnosed by FNB. In addition, we summarized the pathologic and clinical presentations of high grade unclassifiable CUP in which a site of origin or tumor classification could not be identified despite an extensive workup.

## Material and methods

This clinical investigation was conducted in accordance and compliance with guidelines of an institutional Internal Review Board authorization (HSC-MS-19-0303). Liver FNB cases that were performed for liver masses and with a diagnosed of malignant neoplasms during January 2010 – December 2018 were retrieved from the pathology database. Specimens were fixed in 10% neutral buffered formalin and embedded in paraffin for routine histological examination. Immediate assessment for specimen adequacy by cytopathology with touch preparation and Diff-Quick stain was performed for all cases. Cytopathology reports, surgical pathology reports (biopsy and resection if available), and patient medical records, were retrieved and reviewed. Selected cases, including all cases of sarcoma, neuroendocrine tumor, carcinoma of unknown primary, as well as rare tumors such as hepatoblastoma, adrenal cortical carcinoma, paraganglioma, et al. were selected for review. All slides, with a range of 9–56 slides, of selected cases, including cytology (Diff-Quick stain) and surgical pathology (Hematoxylin & Eosin stained and immunohistochemically stained slides), were reviewed.

## Results

A total of 624 cases were identified (Table 1). Metastases (448/624; 71.8%) were much more common than primary liver tumors (176/624; 28.2%). For metastases, adenocarcinoma originating from gastrointestinal tract and pancreas were the two most common neoplasms; while adenocarcinoma from thyroid, prostate, and adrenal cortex were rare in the liver. For primary liver neoplasms, HCC (97/176; 55.1%) was the most common neoplasm, followed by cholangiocarcinoma (73/176; 41.5%) and combined hepatocellular-cholangiocarcinoma (3/176, 1.7%). Rare primary liver neoplasms seen in this study included embryonal sarcoma and hepatoblastoma, both from pediatric patients; and EBV-associated leiomyosarcoma from an adult patient.

**Table 1:** Malignant neoplasms of 624 liver lesions diagnosed by FNB

Cytologic diagnosis	Origin/site/type	Number of cases	Total number
Metastatic adenocarcinoma			317 (50.8%)
	GI tract	143 (45.1%)	
	Pancreatobiliary	74 (23.3%)	
	Breast	44 (13.9%)	
	Lung	24 (7.6%)	
	GYN tract	12 (3.8%)	
	Kidney	12 (3.8%)	
	Prostate	5 (1.6%)	
	Thyroid	1 (0.3%)	
	Adrenal	2 (0.6%)	
Hepatocellular carcinoma^a^			97 (15.5%)
Cholangiocarcinoma^a^			73 (11.7%)
Neuroendocrine neoplasm			58 (9.3%)
	Well-differentiated NETs	16 (27.6%)	
	Poorly-differentiated NECs	42 (72.4%)	
Squamous cell Carcinoma			24 (3.8%)
Lymphoma			17 (2.7%)
	DLBCL	11 (64.7%)	
	Hodgkin lymphoma	3 (17.6%)	
	SLL/CLL	1 (5.9%)	
	Follicular lymphoma	1 (5.9%)	
	Multiple myeloma	1 (5.9%)	
Sarcoma			11 (1.8%)
	Leiomyosarcoma	3 (27.2%)	
	EBV-associated leiomyosarcoma^a^	1 (9.1%)	
	Undifferentiated pleomorphic sarcoma	2 (18.2%)	
	Embryonal sarcoma^a^	1 (9.1%)	
	Malignant SFT	1 (9.1%)	
	Myxoid liposarcoma	1 (9.1%)	
	GIST	2 (18.2%)	
Melanoma			4 (0.6%)
Combined HCC-CC^a^			3 (0.5%)
Urothelial carcinoma			3 (0.5%)
Thymic carcinoma			2 (0.3%)
Hepatoblastoma^a^			1 (0.2%)
Paragnaglioma			1 (0.2%)
Mesothelioma			1 (0.2%)
CUP			12 (1.9%)
Total			624 (100%)

^a^Indicate primary liver malignant lesions

*GI* Gastrointestinal, *GYN* Gynecology, *NETs* Neuroendocrine tumors, *NECs* Neuroendocrine carcinomas, *DLBCL* Diffuse large B cell lymphoma, *SLL/CLL* Small lymphocytic lymphoma/Chronic lymphocytic leukemia, *EBV* Epstein-Barr virus, *SFT* Solitary fibrous tumor, *GIST* Gastrointestinal stroma tumor, *HCC-CC* Hepatocellular-cholangiocarcinoma, *CUP* Carcinoma of unknown primary

In recent years, there has been a significant number of HCC diagnosed by FNB in our institution. Indications for FNB include confirming HCC diagnosis in patients with cirrhosis (63/97, 65%); distinguishing metastasis versus HCC for patients with prior history of malignancy (18/97; 18.6%); distinguishing cholangiocarcinoma or combined cholangiocarcinoma and HCC versus HCC (2/97, 2.1%); diagnosing a liver mass in non-cirrhotic liver (9/97; 9.3%); and determining the primary site of CUP in patients with widespread disease at presentation (4/97; 4.1%). One patient had a history of sarcoidosis and hepatitis C virus-associated cirrhosis. He presented with multiple tumors with calcification. The clinical impression based on imaging was sarcoidosis involving liver; however, biopsy turned out to be HCC.

Neuroendocrine neoplasms (9.3%, 58/624), including well-differentiated neuroendocrine tumors (NETs) and poorly-differentiated neuroendocrine carcinomas (NECs), were among the most common malignant liver tumors. Majority of cases (72.4%, 42/58) were poorly-differentiated NECs, while well-differentiated NETs accounted for 27.6% (16/58) of cases. For poorly-differentiated NECs, small cell (26.2%, 11/42) and large cell carcinoma (4.8%, 2/42) of the lung accounted for 31.0% of these cases (13/42). For well-differentiated NETs, gastrointestinal (GI) tract (81.3%, 13/16) was the predominant site of origin.

Metastatic squamous cell carcinoma was identified in 3.8% (24/624) of case. The most common primary sites were uterine cervix (29.2%; 7/24), followed by head and neck, (25.0%; 6/24), esophagus (16.7%; 4/24), lung (8.3%, 2/24)), penile (4.2%, 1/21), anus (4.2%, 1/21), and pancreatobiliary (4.2%, 1/24). The primary sites for the remaining two cases were undetermined (8.3%, 2/24).

Sarcoma (11/624; 1.8%) was uncommon compared with carcinoma. In our study, there were three cases of metastatic leiomyosarcoma (two patients with history of uterine leiomyosarcoma, the third 84-year-old patient had remote history of hysterectomy and bilateral salpingo-oophorectomy but no leiomyosarcoma diagnosis) and one case of primary EBV-associated leiomyosarcoma in a Human Immunodeficiency Virus (HIV) - positive patient. Other sarcomas that metastasized to the liver include gastrointestinal stroma tumor (GIST), undifferentiated pleomorphic sarcoma (UPS), malignant solitary fibrous tumor (SFT), myxoid liposarcoma, and primary embryonal sarcoma from a pediatric patient.

**Fig. 1:**
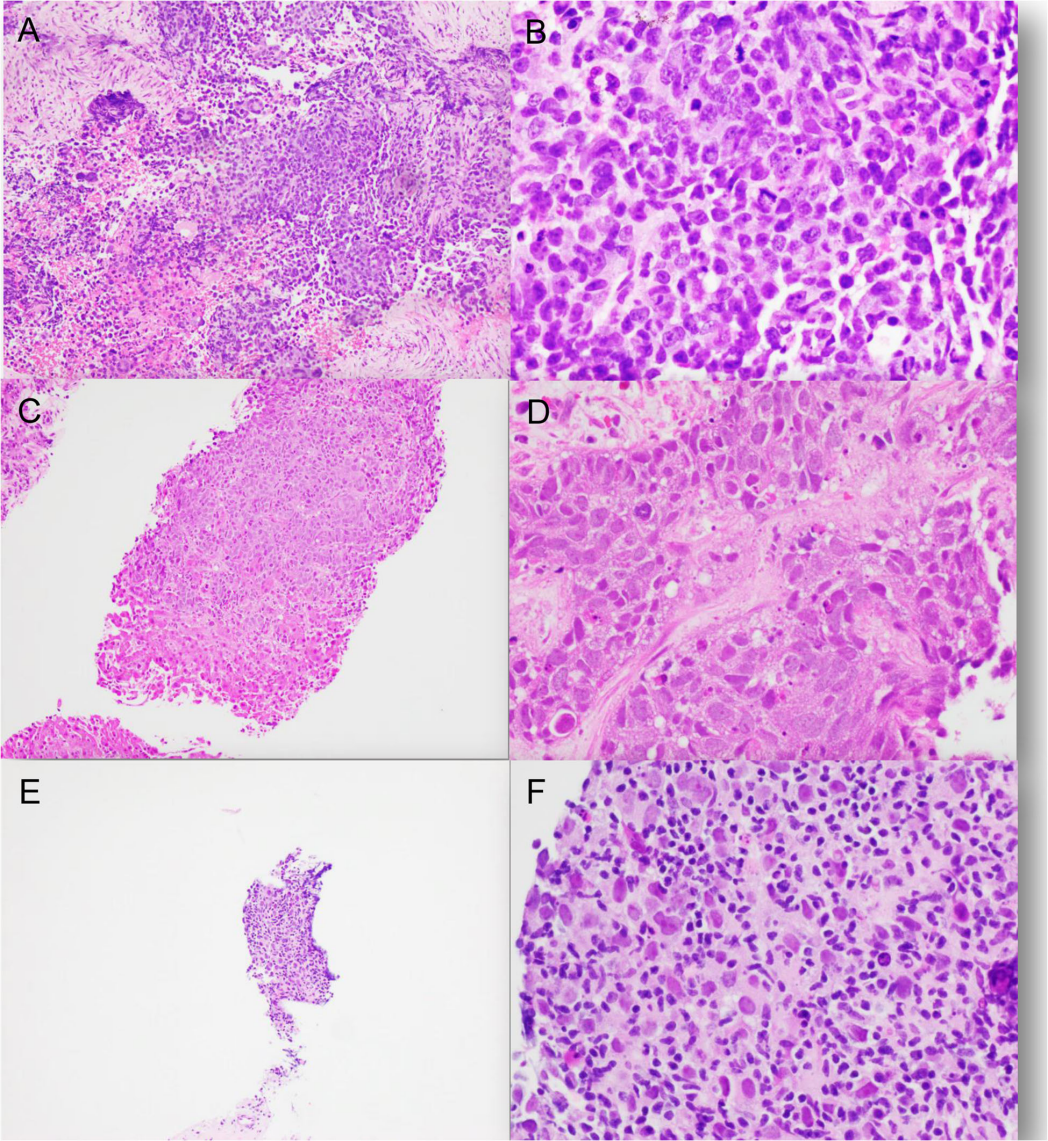
**a** and **b**: Unclassifiable high grade small blue cell tumor. Tumor cells have high nuclear to cytoplasmic ratio (N/C ratio), brisk mitotic and apoptotic activity. Multinucleated giant tumor cells are also seen. Tumor cells are focally positive for pancytokeratin. Lymphoma, neuroendocrine, melanoma, skeletal muscle, and lineage specific markers are all negative (not shown). Fluorescence in-situ hybridization for EWSR1 gene is negative. (A 100x; B 400x). **c** and **d**: Unclassifiable large eosinophilic cell tumor. Tumor cells have a moderate amount of eosinophilic cytoplasm, fine chromatin and inconspicuous nucleoli. Tumor cells are positive for CK7. Neuroendocrine, melanoma, hepatocellular, kidney, adrenal and other lineage specific markers are all negative. (C 100x; D 400x). **e** and **f**: Unclassifiable neoplasm due to scant material. Cell block preparation shows one cluster of tumor cells with nuclear pleomorphism and abundant lymphoid infiltration. Limited immunohistochemical stains were performed due to limited cellularity. Tumor cells are positive for AE1/AE3, and negative for HepPar-1, arginase, TTF-1, NKX3.1, PAX8, CK7, and CK20

Twelve (1.9%, 12/624) cases were diagnosed as carcinoma or high grade malignancy favor carcinoma, of unknown primary (CUP), due to lack of specific protein expression or limited biopsy tissue. The primary site could not be determined both clinically and pathologically. Patient’s ages ranged from 31 to 81 years. Male patients were more common than female patients (9: 3). The majority of patients presented with wide spread disease involving multiple organs, including liver, lung, lymph nodes, bone, and others (Table 2). Two patients had a history of malignancy, however the histomorphological as well as immunohistochemical characterization of the liver masses were different from the patients’ known malignancies. Morphologically, 3 cases were high grade small blue cell tumor; 3 cases are high grade large eosinophilic cell tumor; 2 cases were high grade adenocarcinoma; 1 case had spindle cell morphology; the remaining 3 cases were unclassifiable due to scant cellularity (Fig. 1). All twelve cases showed pleomorphic tumor cells with brisk mitotic and apoptotic activity, and large areas of necrosis are present in majority of these cases. For small blue cell tumor, differential diagnosis included poorly differentiated neuroendocrine carcinoma, basaloid squamous cell carcinoma, lymphoma, sarcoma, and melanoma. For large eosinophilic neoplasm, differential diagnosis includes carcinoma from thyroid, liver, kidney, and adrenal glands, as well as melanoma and sarcoma. For spindle cell malignancy, differential diagnosis includes spindle cell carcinoma and sarcoma. Extensive immunohistochemical workups were performed, except for cases without enough material. The tumor cells were all positive for pancytokeratin (AE1/AE3) and/or CK7, while other lineage specific markers including TTF-1, CDX2, PAX8, GATA3, synaptophysin, chromogranin, p40, CK5/6 et al. were all negative. Molecular testing was performed only for one case (case11). Fluorescent in-situ hybridization (FISH) for *EWSR1 *gene was performed for this case to rule out desmoplastic small round cell tumor for this 31-year-old patient, which showed no evidence of *EWSR1 *gene translocation. Given the AE1/AE3 and/or CK7 positivity and widespread disease involving lung, liver and other organs, all these cases were diagnosed as carcinoma or favor carcinoma. Prognosis was poor for these patients (Table 2). More than half of these patients gave up treatment or received palliative care only.

**Table 2:** Clinical and pathologic presentations of the CUP cases

Case	Age	Gender	Imaging finding	Pertinent history	Clinical impression	Pathologic finding	Positive IHC	Negative IHC (selected pertinent negatives are listed)	Treatment	Prognosis
1	81	M	Multiple liver and lung masses	Smoking	Stage IV lung cancer	Large eosinophilic cell tumor	AE1/AE3, CK7	TTF-1, Napsin A, Synaptophysin, Chromogranin, HepPar-1, Arginase	Discharged to hospice care	Lost follow up
2	62	F	Multiple liver masses	Rectal squamous cell carcinoma, s/p surgery and chemo-radiation	Recurrent rectal carcinoma	Blue cell tumor	AE1/AE3, CK7	p40, p63, CK5/6, Synaptophysin, Chromogranin	Palliative chemotherapy	DOD 14 months
3	64	M	Multiple liver masses, abdominal and retroperitoneal lymphadenopathy	None	CUP	Blue cell tumor	CK7	CK20, HepPar-1, Arginase, Synaptophysin, Chromogranin	Palliative care	DOD 1 months
4	42	M	Multiple liver masses, retroperitoneal lymphadenopathy	Childhood seminoma diagnosed at age 12, recurred at age 22	Recurrent seminoma	Poorly differentiated adenocarcinoma	CK7	OCT3/4, CD117, CD30, PLAP, Glypican-3, CK20, CDX2, TTF-1, GATA3	Palliative care	DOD 5 weeks
5	47	M	Multiple liver, lung, and spleen masses	Smoking	CUP	Large eosinophilic cell tumor	CK7	CK20, TTF-1, Napsin A, HepPar-1, PAX8, CDX2, MelanA	Discharged to hospice care	Lost follow up
6	56	F	Multiple liver and bilateral ovarian masses, intraabdominal and mesenteric nodules	Liver cirrhosis	Mesothelioma vs. ovarian carcinoma	Poorly differentiated adenocarcinoma	CK7, Bap-1 retained	CK20, PAX8, GATA3, ER, PR, TTF-1, WT-1	Chemo-radiation	DOD 10 months
7	59	F	Multiple liver masses, multiple lytic bone lesions	Liver cirrhosis	CUP	Scant pleomorphic tumor cells	CK7	CK20, TTF-1, Glypican 3, HepPar-1, CDX2	Discharged to hospice care	Lost follow up
8	77	M	Multiple liver and lung masses, and bone lytic lesions	None	Stage IV lung cancer	Spindle cell carcinoma	CK7	CK20, TTF-1, Napsin A, p40, CK5/6	Scheduled for oncology appointment, lost follow up	AWD 5 weeks
9	49	M	Multiple lung, liver, brain masses	Smoking and COPD	CUP	Scant pleomorphic tumor cells	CK7	Heppar-1, CK5/6, TTF-1	Palliative care	DOD 1 week
10	47	M	Unknown Imaging study performed at outside hospital	None	Unknown	Large eosinophilic cell tumor	AE1/AE3	HepPar-1, TTF-1, CDX2, Synaptophysin, Chromogranin	Unknown	DOD 3 years
11	51	M	Multiple liver and lung masses, multiple abdominal lymphadenopathy, and omentum nodules	Liver cirrhosis	CUP	Scant pleomorphic tumor cells	AE1/AE3	CK7, CK20, HepPar1, TTF-1, Synaptophysin, Chromgranin, CD45, S100	Palliative care	DOD 3 weeks
12	31	M	Multiple liver masses, large abdominal masses	None	Cholangiocarcinoma	Blue cell tumor	AE1/AE3	SOX10, S100, Chromogranin, Synaptophysin, HepPar1, Desmin, Myogenin, CD43, CD45, Arginase,WT1, CD34, OCT3/4, CK19	Palliative care	AWD 3 weeks

*M* Male, *F* Female, *CUP* Carcinoma of unknown primary, *AWD* Alive with disease, *DOD* Die of disease, *COPD* chronic obstructive pulmonary disease

## Discussion

FNB is commonly used for the diagnosis of mass lesions in the liver. In majority of these cases, clinical information and imaging studies can provide valuable information for diagnosis. For primary neoplasms, HCC is usually suspected in cirrhotic liver, while cholangiocarcinoma is more common in non-cirrhotic liver [16]. For metastatic disease, the vast majority of patients have an established diagnosis of malignancy in the primary site. However, not uncommonly, patients may present with liver metastasis without a known diagnosis. Given the tremendous diversity of neoplasms that can occur in the liver, diagnosis can be challenging, especially for tumors that are rare in the liver or when FNB tissue is limited. In these cases, imaging studies are often helpful. In our study, the majority of patients who presented with liver metastases and without an established diagnosis of malignancy, imaging study revealed a bulky mass at the primary site. Only in a minority of patients with widespread disease were the primary sites not obvious by imaging. Morphologic assessment with judicious use of immunohistochemical stains can help to identify the primary sites in many of those cases, especially with advanced immunohistochemical techniques and many recently developed lineage-specific transcription factors.

A tremendous variety of malignant neoplasms can occur in the liver, with metastases being much more common than primary neoplasms [1–4]. Tumors from almost the entire human body can metastasize to the liver. The most common primary sites include gastrointestinal tract, pancreatobiliary tract, breast, lung, and gynecological tract. We found that adrenocortical carcinoma (2 cases), thymic carcinoma (2 cases), urothelial carcinoma (3 cases), mesothelioma (1 case), and thyroid carcinoma (1 case) are rare in the liver (Table 1). In this study, there was also one case of malignant paraganglioma metastatic to the liver in a patient with a history of retroperitoneal paraganglioma (Table 1). Diagnosis for these rare tumors can be challenging. One of the adrenocortical adenocarcinomas was originally diagnosed as “oncocytic neoplasm”. The tumors cells had abundant clear to eosinophilic cytoplasm with nested and trabecular growth pattern. Imaging showed a large necrotic mass in the upper abdominal cavity. Additional immunohistochemical stains showed that the tumor cells were positive for Melan A and inhibin, supporting a diagnosis of adrenocortical carcinoma. One urothelial carcinoma was originally diagnosed as “high grade carcinoma with squamous differentiation”. The patient later was found to have a bladder mass with positive urine cytology. Familiarization with histomorphologic features of these rare neoplasms is important for correct diagnosis.

**Fig. 2:**
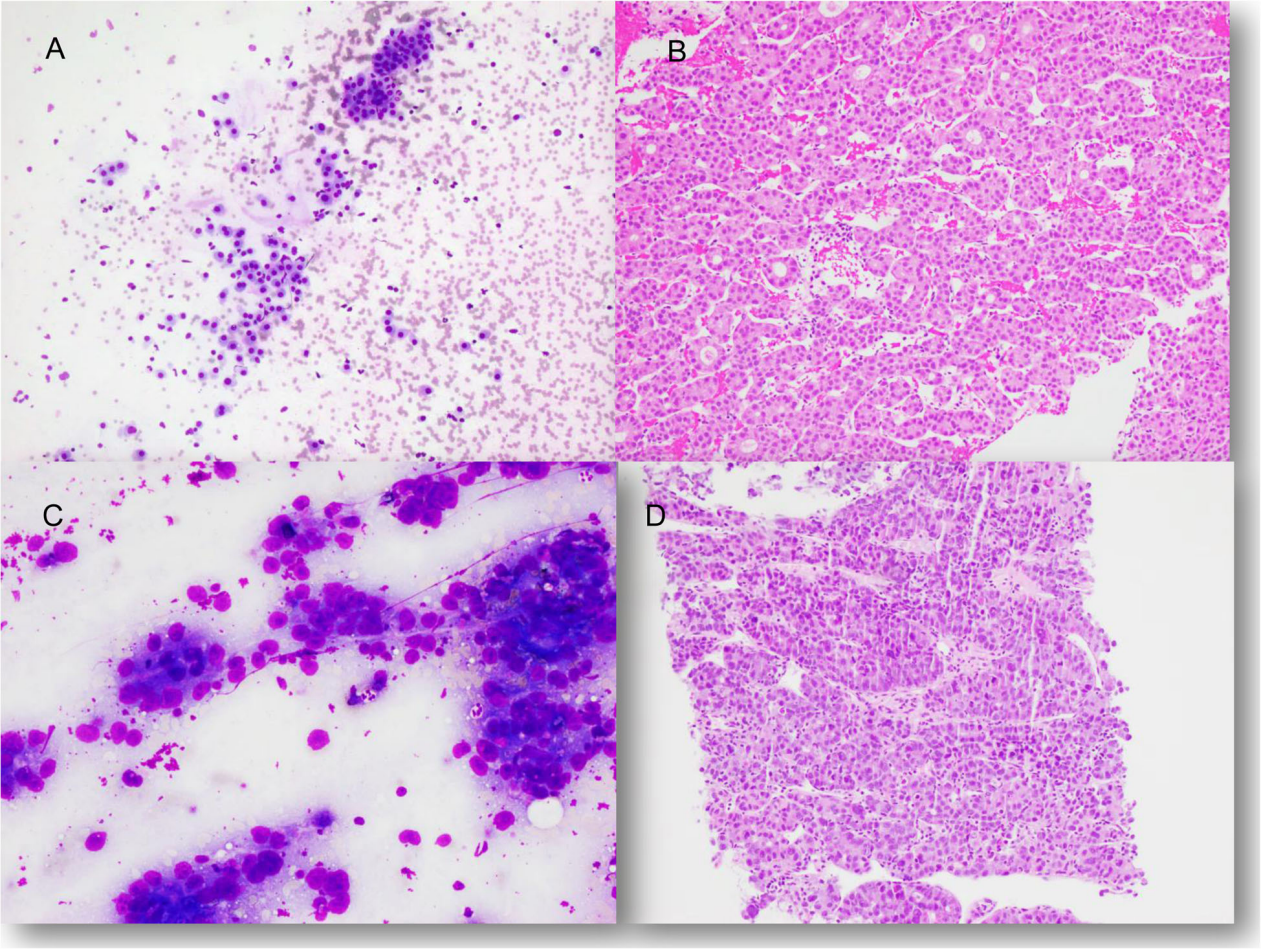
**a** (Diff-Quik stain, 200x) and **b** (H&E, 100x): Well-differentiated HCC demonstrate monotonous tumor cells with slight hyperchromasia, discohesiveness, and slightly increased N/C ratio on cytologic preparation. Concurrent liver biopsy shows pseudoglandular formation. **c** (Diff-Quik stain, 200x) and **d** (H&E, 100x): Poorly differentiated HCC shows prominent nuclear atypia, many naked nuclei and prominent nucleoli on cytologic preparation. Concurrent biopsy shows a high grade neoplasm with brisk mitotic activity

A significant numbers of HCC were diagnosed by FNB in our practice in recent years, including HCC with typical imagining findings. In addition to histologic confirmation of an HCC diagnosis, FNB can provide valuable information about tumor grading (Fig. 2), which is closely related to patient survival. FNB is also essential for molecular profiling which is potentially useful for targeted therapy in the era of personalized medicine [20]. The pathogenesis of HCC comprises a multistep process that involves genetic and epigenetic events of multiple genes. Multiple kinase inhibitors and monoclonal antibodies have shown efficacy in treating HCC in clinical trials. A molecular characterization of HCC is necessary to identify patient subclasses according to drug sensitivity, which will lead to a more effective treatment [20].

CUP is one of the most common malignancy, accounting for up to 5% of new cancer diagnosis [21]. The incidence of CUP is highest in patients between 60 and 75 years of age. Multiple metastases at presentation involving liver, abdomen, brain, thorax, bones and lungs are common. Prognosis is dismal with median survival of less than 1 year, despite multimodal therapies including combined surgery, chemotherapy, radiotherapy and targeted molecular therapy. Histopathologically, the most common CUPs include adenocarcinoma, undifferentiated carcinoma, squamous cell carcinoma, and neuroendocrine carcinoma. Undifferentiated carcinoma accounts for 30% of all CUP cases, and these patients show the worst prognosis compared with other types of CUP [21]. In this study, we identified 12 cases of undifferentiated CUP. Morphologically, all were high grade neoplasms with sheets, nests or single file growth patterns and no obvious morphologic differentiation (Fig. 2). All cases were only positive for cytokeratin, while lineage specific markers were all negative. These cases were diagnosed as poorly differentiated carcinoma/adenocarcinoma, or undifferentiated malignant neoplasm favor carcinoma. As the primary site could not be identified, no site-specific treatment could be offered and these patients generally had poor outcomes (Table 2). For these unclassifiable neoplasms, little is known about etiology, clinical behavior, or the optimal treatment. Recent studies have found that these unclassifiable neoplasms represent a heterologous group of tumors [18]. Many specific entities have been recently identified with new molecular and/or immunohistochemical tools, especially in the head and neck region [21–25]. Identification of these new entities is important for us to understand the epidemiology, etiology, tumor behavior, and most importantly, identify effective patient treatment [26]. Importantly, diffuse strong keratin positivity can be seen in some sarcomas, such as Adamantinoma-like Ewing sarcoma and desmoplastic small round cell tumor. Familiarization with the morphologic features of these entities and confirmation with molecular testing are important for correct diagnosis. With the ever-growing advances in targeted therapy in the era of personalized medicine, accurate classification and diagnosis is essential for identifying the appropriate treatment for each individual patient [23, 27]. Future study is necessary to reveal the molecular and clinicopathologic features of these unclassifiable CUP cases.

## Data Availability

Not applicable.

## Abbreviations

FNB: Fine-needle biopsy
CUP: Carcinoma of unknown primary
HCC: Hepatocellular carcinoma
